# Decreased abundance of Akkermansia after adrenocorticotropic hormone therapy in patients with West syndrome

**DOI:** 10.1186/s12866-021-02189-z

**Published:** 2021-04-23

**Authors:** Lu Xu, Dandan Chen, Congying Zhao, Lihua Jiang, Shanshan Mao, Chao Song, Feng Gao

**Affiliations:** Department of Neurology, The Children’s Hospital, Zhejiang University School of Medicine, National Clinical Research Center for Child Health, Hangzhou, 310003 Zhejiang China

**Keywords:** West syndrome, Gut microbiota, ACTH therapy, Akkermansia

## Abstract

**Background:**

Infants suffer from a severe epileptic encephalopathy known as West syndrome (WS). Treatment with adrenocorticotropic hormone (ACTH) indicates the involvement of the gut-brain axis in WS. Several pieces of evidence show the communication of the gut microbiota (GM) with the brain via the hypothalamic–pituitary–adrenal axis (HPA axis) and blood cytokines. This study aimed at (1) determining the GM diversity in infants having WS and (2) comparing the results of infants having WS with those of the healthy infants and also in the patients with WS before and after the ACTH therapy.

**Results:**

In this study, 29 infants with WS and 29 healthy infants aged 3–13 months were recruited. Fecal samples were collected, and DNA was extracted and sequenced on the Illumina MiSeq platform. Kruskal-Wallis rank-sum test was used to analyze the between-group differences in the Chao1 index, Shannon index, and the abundances of GM at different taxonomy levels. R software was used to plot the graphs. The top five dominant GM genera between patients with WS and healthy infants showed no significant differences. However, the relative abundance of genus Akkermansia was observed to be significantly (*P* = 0.011) higher in the BT group than in the HC group and AT group. After 2 weeks of ACTH therapy, the relative abundance of Akkermansia significantly (*P* = 0.003) decreased.

**Conclusion:**

The relative abundance of Akkermansia was observed to be significantly higher in patients with WS than that in healthy infants. However, the relationship between Akkermansia and WS pathogenesis needs to be clarified in further studies.

## Background

Epilepsy is a disorder in which there is an abnormal discharge of neurons in the cerebral cortex, which causes excitability and inhibitory disorders of the brain network. A severe epileptic encephalopathy observed in infants is known as the West syndrome (WS), which has an onset at the age of 3 months to 2 years and an incidence of 0.25 to 0.42 per 1000 lives. WS is a challenge to the pediatric neurologists investigating the mechanism and treatment. It is characterized by infantile spasms, hypsarrhythmia (as recorded on interictal electroencephalographs [EEGs]), and neurodevelopmental delay. Conventional anti-epileptic drugs are ineffective in the treatment of WS. In guidelines and some reviews, adrenocorticotropic hormone (ACTH), vigabatrin, and oral corticosteroids are recommended as the first-line drug treatment for WS [1–3].

ACTH therapy is a highly effective treatment for infantile spasms and is widely accepted among pediatricians. However, the underlying mechanism of ACTH in the treatment of WS remains unknown. Since epilepsy is a broad and complex disease, whether there is a relationship between the gut microbiota and WS as a representative epilepsy syndrome should be analyzed.

The communication between gut microbiota (GM) and the brain involves the vagus nerve, HPA axis, gut immune system, and neurotransmitters synthesized by intestinal bacteria [1]. Further studies on the brain–gut–microbiota axis have shown the involvement of various regions of the nervous system. Earlier studies have mainly focused on psychiatric and neurological pathologies, including chronic pain [2], autism spectrum disorder [3], Parkinson’s disease [4], and multiple sclerosis [5].

Recent studies have shown that GM plays an important role in the treatment of refractory epilepsy, especially in mediating the antiseizure effects of the ketogenic diet [6, 7]. In mouse models of ketogenic diet, the effects of anti-epileptic seizures are mainly mediated by the GM through the modulation of hippocampal γ-aminobutyric acid/glutamate ratio. Another study reported the biotransformation of the anti-epileptic drug, zonisamide, by the GM [8], demonstrating the intestinal bacteria playing a major role in the reductive metabolism of zonisamide to 2-sulphamoylacetylphenol in-vivo.

The communication between the GM and the brain via the gut-brain axis might influence WS. In this study, the presence of GM genera in patients with WS was investigated. The differences in the GM between the patients with WS and the healthy infants and in patients before and after short-term ACTH treatment were evaluated to understand the underlying mechanisms of ACTH therapy in WS.

## Results

### Clinical improvement after ACTH therapy

A total of 29 (M:F = 20:9) infants with WS as patients and 29 healthy infants (M:F = 19:10) as controls (HC), all aged 3–13 months, were evaluated (Table 1). Patients with WS were assessed before (BT) and after (AT) the ACTH therapy. After the treatment with ACTH, 15 (51.7%) patients (M:F = 10:5) were spasm-free, 12 (M:F = 8:4) (42.1%) patients had a 60–90% reduction in seizure frequency, and two patients (M:F = 2:0) were unresponsive. However, the EEG results showed that 7 (M:F = 4:3) (21.1%) patients had persistent hypsarrhythmia at the end of treatment; 1 patient had no follow-up EEG.

**Table 1:** Characteristics of patients with West Syndrome (WS)

Patient NO.	Etiology	Feeding patterns	Anti-seizure drugs	Clinical outcome	EEG(hypsarrhythmia) after treantment	Fecal sampling time point(the day of ACTH treatment)
1	TSC	AF	None	Spasm-free	Relieved	14
2	Unknown	BF	LEV、TPM、CZP	No response	Persisted	14
3	Unknown	AF + CF	VPA、TPM	Spasm-free	Resolved	15
4	Unknown	AF + CF	None	Relieved	Persisted	14
5	Unknown	CF	None	Relieved	Relieved	15
6	HIE	AF + CF	None	Spasm-free	Persisted	14
7	TSC	CF	None	Relieved	Relieved	14
8	Unknown	BF + CF	TPM、NZP	Relieved	Relieved	14
9	Unknown	BF + CF	VPA、NZP	Spasm-free	Relieved	9
10	HIE	AF	VPA	Relieved	–	14
11	Unknown	BF + CF	None	Relieved	Relieved	15
12	Unknown	MF	VPA、TPM	Relieved	Relieved	14
13	NF1	AF + CF	VPA、TPM、LEV	Spasm-free	Resolved	10
14	FCD	MF	None	Spasm-free	Persisted	14
15	Unknown	BF	VPA、OXC、LEV	Relieved	Relieved	14
16	Unknown	MF	None	Spasm-free	Relieved	14
17	Unknown	CF + MF	None	Spasm-free	Relieved	14
18	Unknown	MF	None	Spasm-free	Relieved	15
19	CCM	AF	VPA	Spasm-free	Relieved	8
20	Unknown	BF	None	Spasm-free	Relieved	14
21	Unknown	MF	TPM	Spasm-free	Resolved	14
22	Unknown	MF	None	Relieved	Persisted	14
23	Unknown	MF	None	Relieved	Persisted	14
24	HIE	AF	None	No response	Relieved	14
25	Unknown	MF	TPM	Spasm-free	Resolved	14
26	TSC	AF	TPM	Spasm-free	Relieved	14
27	Unknown	BF + CF	None	Relieved	Persisted	14
28	Unknown	AF	None	Spasm-free	Relieved	14
29	Unknown	AF	None	Relieved	Relieved	14

*AF* artificial feeding, *BF* breast feeding, *CF* complementary feeding, *CCM* congenital cleft malformation, *CZP* clonazepam, *EEG* electroencephalograph, *F* female, *FCD* focal cortical dsysplasia, *HIE* Hypoxic-ischemic encephalopathy, *LEV* levetiracetam;M,male, *MF* mixed feedin, *NF1* Neurofibromatosis 1, *NZP* nitrazepam, *OXC* oxcarbazepine, *TPM* topiramate, *TSC* Tuberous sclerosis, *VPA* valproic acid. All patients had hypsarrhythmia on electroencephalography

Three patients (two seizure-free and the one with a 60–90% reduction in seizure frequency) required antibiotics due to respiratory infection caused by low immunization. The fecal samples were collected before the antibiotic treatment (before the end of the planned two-week treatment period). For the remaining patients with WS, fecal sample collection was completed on Day 14 or 15. Seventeen (58.6%) patients with WS were not given anti-epileptic drugs before the study.

### Differences in GM among patients with WS and HCs

On average, there were 34,853 high-quality reads on each sample after filtration and quality control. The reads were clustered into operational taxonomic units (OTUs) with a similarity index of 97%, with the most abundant reads as the representative reads. The OTUs were further assigned to certain taxa by rdp-classifier (V1.12). Subsequently, profiling tables at different levels were acquired by Qiime (V1.9).

**Fig. 1:**
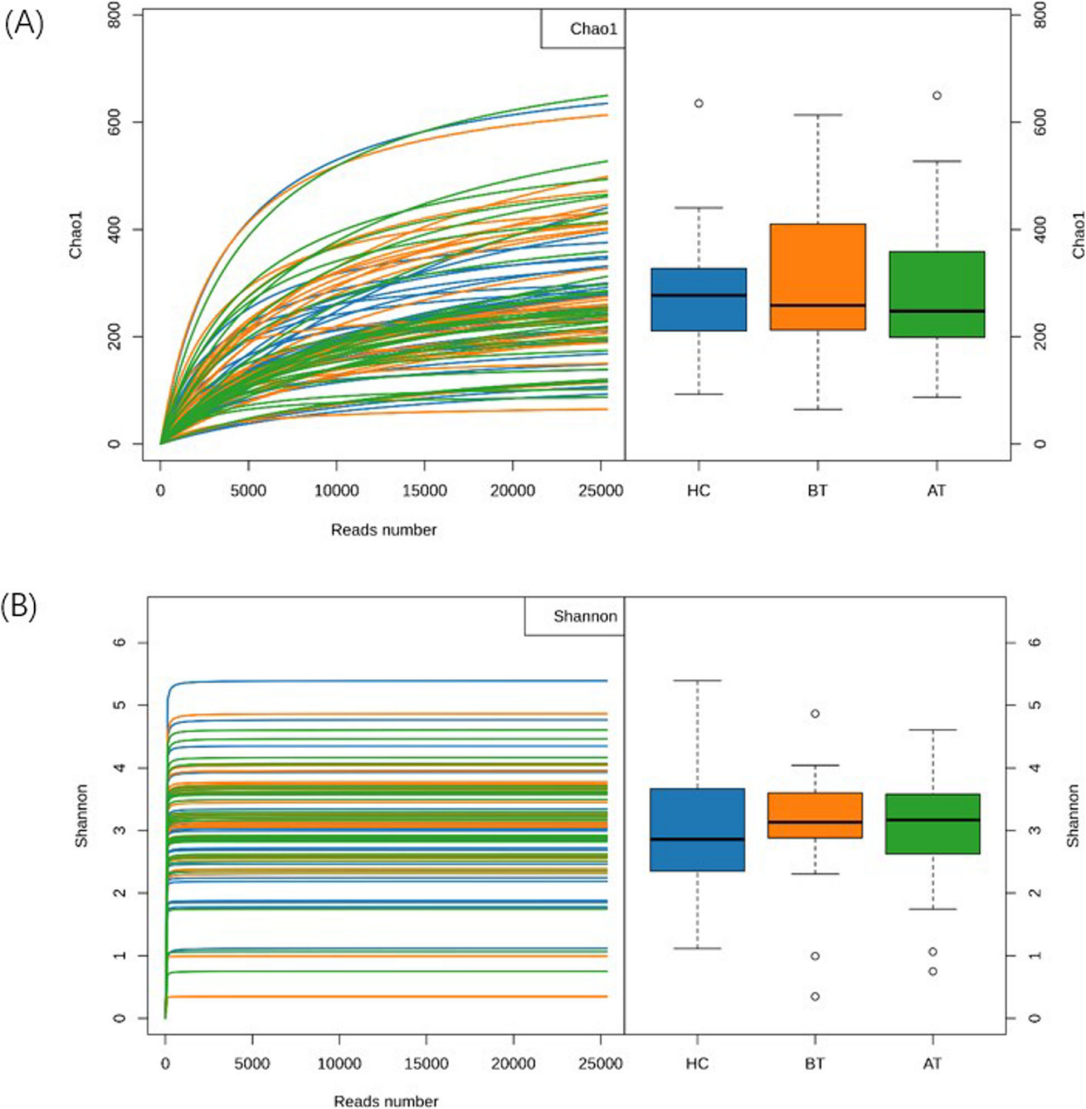
Gut microbiota Chao1 index and Shannon index(α diversity) among healthy infants (HC;blue), patients with WS before therapy (BT; orange) and after therapy (AT; green). **a** Chao1 index shows the number of possible taxa; **b** Shannon index shows how even the distribution of taxonomy composition. There was no difference in both chao1 index (*P* = 0.90, calculated by the Kruskal-Wallis Test) and Shannon index (*P* = 0.61) among HC, BT and AT groups

**Fig. 2:**
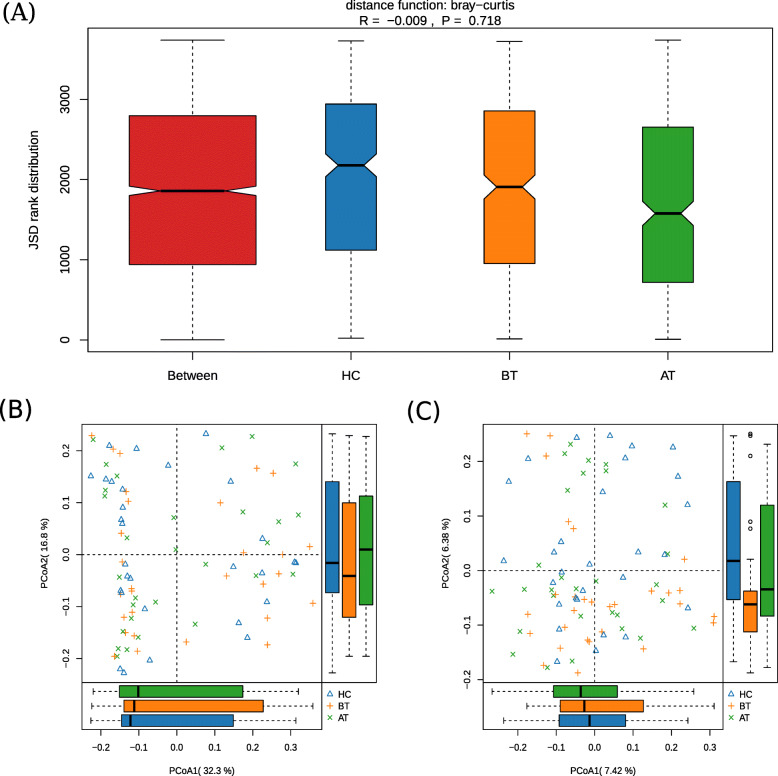
β diversity of gut microbiota in three groups. **a** analysis of similarity based on Bray-Curtis distance. **b** The principal coordinate analysis (PCoA) (*p* = 0.62) based on weighted Unifrac distances showed that gut microbiota of three groups had no significant alteration in proportion, and **c**. PCoA2(*P* = 0.03) on unweighted Unifrac showed species of gut microbiota in BT group were different from those in the other two groups

**Fig. 3:**
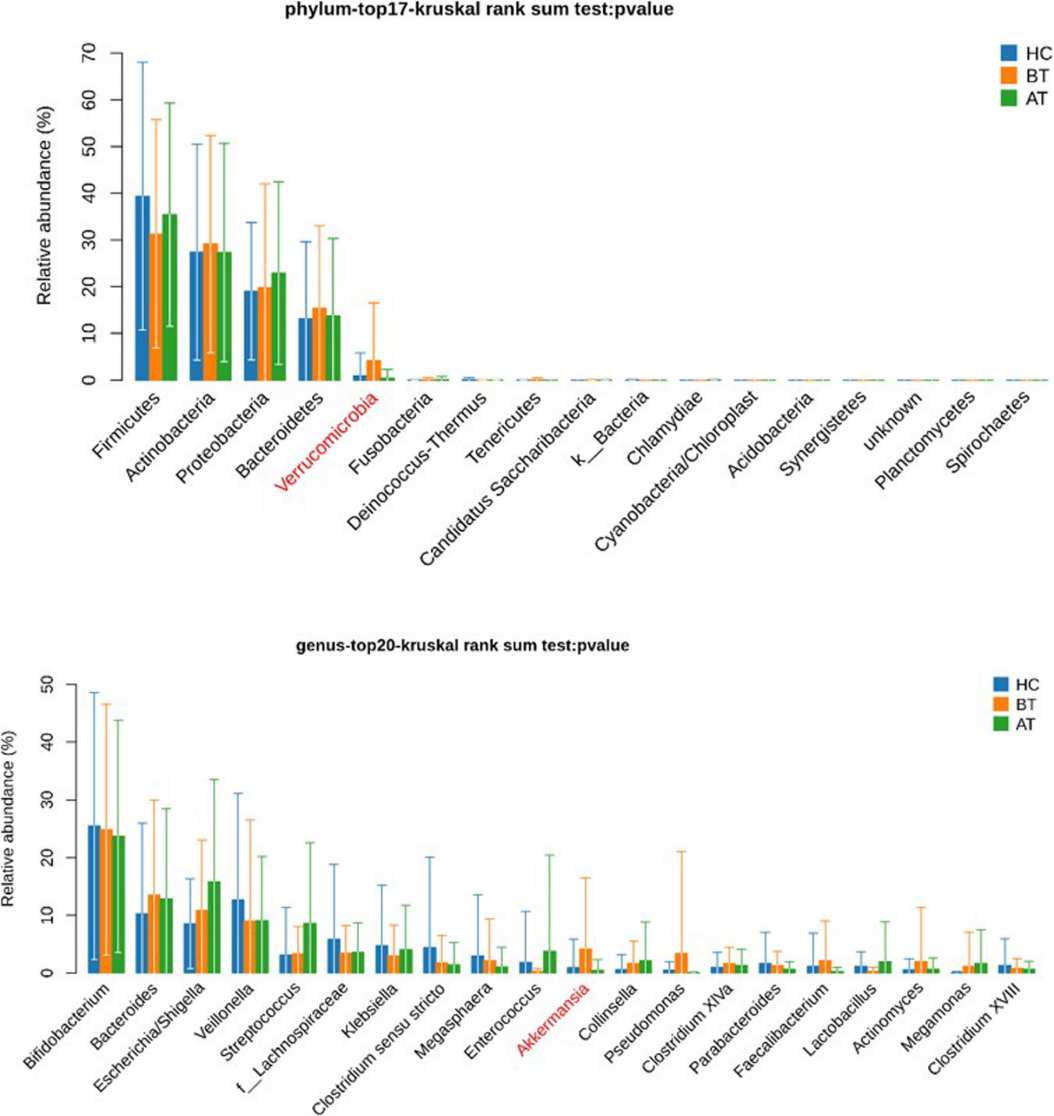
Differences analyzed at phylum and genus level of microbiota among three groups. The relative abundance of Verrucomicrobia at phylum level and Akkermansia at genera level of BT group showed significant differences from same microbiota of the other two groups

The alpha diversity indices (Chao1 and Shannon) varied widely within the three groups (AT, BT, and HC), which resulted in range overlaps between the groups and, therefore, no statistical significance was observed (Kruskal-Wallis rank-sum test, *P* = 0.90 for Chao and *P* = 0.61 for Shannon) (Fig. 1a and b). The differences in the microbial communities were estimated by the analysis of similarity (anosim) and principal coordinates analysis (PcoA). The wide distribution of Bray–Curtis distance within the groups (Fig. 2a) demonstrated that WS might not be strongly related to the whole microbial community. Furthermore, the first components of PcoA (PcoA1) (Fig. 2b and c), based on weighted and unweighted UniFrac distances, did not reveal any difference among the three groups. Only the second component of unweighted-UniFrac-based PcoA showed a divergence, where the group BT diverged from the other two groups (Kruskal-Wallis rank-sum test, *P*-value = 0.03). The top five phyla observed in the three groups were Firmicutes, Actinobacteria, Proteobacteria, Bacteroidetes, and Verrucomicrobia (Fig. 3), with only Verrucomicrobia showing significant difference in the Kruskal–Wallis rank-sum test (*P* = 0.01). At the genus level, the relative abundance of Akkermansia was significantly higher (*P* = 0.01, relative abundance x ± sd: 0.92% ± 4.94% for HC; 4.19% ± 12.3% for BT; 0.44% ± 1.87% for AT). The significance of Verrucomicrobia must result from the fact that Akkermansia belongs to the phylum Verrucomicrobia. Further paired tests confirmed that Akkermansia was significantly higher in BT than the other two groups (for HC–BT comparison, Wilcoxon rank-sum test, *P* = 0.03; for BT–AT comparison, Wilcoxon signed rank test, *P* = 0.003). The dietary pattern and medication used in the AT and BT groups was kept the same during the treatment. However, after the ACTH therapy, the relative abundance of Akkermansia dramatically decreased.

**Fig. 4:**
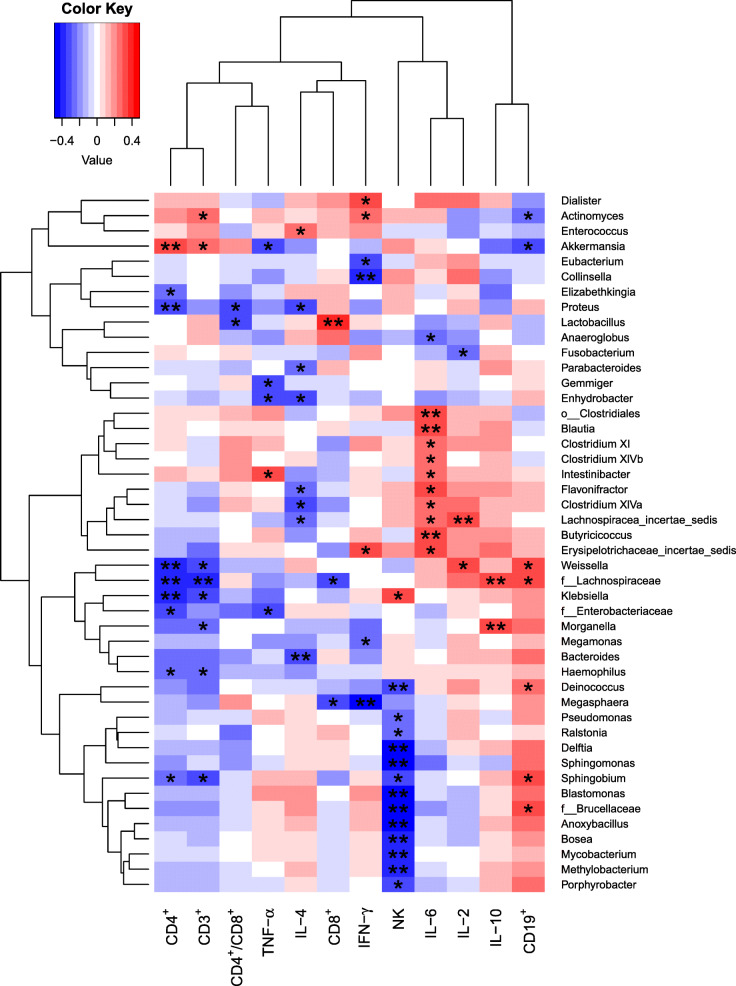
Heatmap for Spearman’s rank-sum correlation coefficient between genera and some immune-factors (immunocytes and cytokines). The tree on the left shows the clustering of genera, while the one on the top shows the clustering of immune-factors. The red color on the heatmap indicates a positive correlation, while blue indicates a negative correlation. The darker the color, the larger the correlation coefficient. *: *p*-value ≤0.05, **: *p*-value ≤0.01

Spearman’s rank-sum correlation coefficient test was used to evaluate the correlation between the genera and some cytokines (TNF-α, IL-4, IFN-γ, IL-6, IL-2, and IL-10) and immunocytes (CD3^+^, CD4^+^, CD8^+^, CD19^+^, CD4^+^/CD8^+^, and NK) in the patients before (AT group) and after (BT group) the treatment (Fig. 4). A total of 46 genera were observed to be significantly correlated to at least one of the cytokines or immunocytes. Akkermansia was positively correlated to CD3^+^ and CD4^+^, with Spearman’s rank-sum coefficients of 0.283 and 0.346, respectively; however, it was negatively correlated to TNF-α and CD19^+^, with Spearman’s rank-sum coefficients of − 0.326 and − 0.338, respectively.

**Fig. 5:**
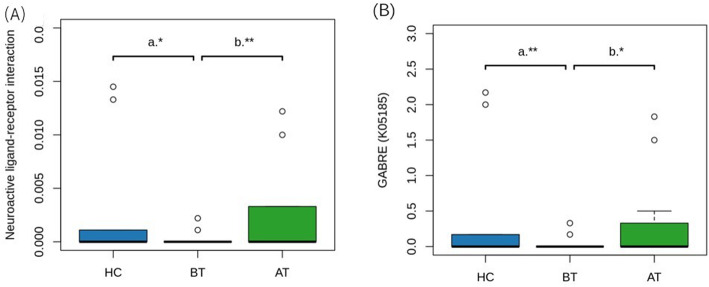
**a** Boxplot of the abundance of KEGG pathway “Neuroactive ligand-receptor interaction”. **b** KO analysis shows GABRE (K05185, γ-aminobutyric acid receptor subunit epsilon), is significantly low in BT group. *P* value: ** ≤ 0.01, * ≤ 0.05; a indicates Wilcoxon rank sum test; b indicates Wilcoxon signed rank test

KEGG (Kyoto Encyclopedia of Genes and Genomes) pathway abundances were predicted by Picrust2, get pathway, and KO (KEGG Orthology) profiling. Wilcoxon’s rank-sum test showed that two pathways were significantly abundant: 1) Neuroactive ligand-receptor interaction (ko04080); 2) Meiosis-yeast (ko04113). The first one is really interesting, as shown in the figure (Fig. 5a). Furthermore, the KO analysis showed that GABRE (K05185, γ-aminobutyric acid receptor subunit epsilon) was significantly low in BT (Fig. 5b).

## Discussion

The existence of a bidirectional relationship between the GM and the brain, termed as the gut–brain–axis, has been well evidenced and is also associated with numerous health disorders [2–5]. The recent findings show that GM impacts the neuronal activity leading to the development of epilepsy [6]. A specific epilepsy syndrome, known as WS, was selected to compare the GM diversity between the patients and HCs, and the GM before and after treatment with ACTH.

The results of this study showed no differences in the relative abundances of the top five genera (Bifidobacterium, Bacteroides, Escherichia/Shigella, Veillonella, and Streptococcus; Mean relative abundance≥10%) between the patients with WS and the HCs. This indicates that there is no obvious GM dysbiosis in the patients with WS. However, the relative abundances of Verrucomicrobia at the phylum level and Akkermansia at the genus level were significantly higher in the patients with WS than those in the HC group. Interestingly, ACTH is believed to regulate antibody synthesis through the adrenal cortex. Combined with the fact that most of the gut flora have a mutualistic interaction with humans, if ACTH takes effect through antibody, it will influence few taxa, which seems to be consistent with our results. As we know, various factors influence GM, including geography, diet, stress, lifestyle, socioeconomic factors, exercise, physiological stress caused by critical illness, and medications such as agents that cause gastric acid suppression and antibiotics [9]. Finally, the results showed that the relative abundance of Akkermansia was significantly and dramatically decreased after the treatment with ACTH.

In the human GM, Akkermansia (phylum Verrucomicrobia) is present abundantly in normal conditions, although in certain conditions, such as glucose metabolism, obesity, and inflammatory bowel disease, its relative abundance is decreased [10, 11]. Akkermansia is reported to be beneficial for host health and is considered a promising probiotic candidate [12–14] as it facilitates gut barrier integrity, increases mucus thickness, and is beneficial for metabolism and immune responses [15]. Plovier H et al. demonstrated that a specific protein (Amuc_1100) isolated from the outer membrane of Akkermansia, interacting with Toll-like receptor 2, could improve the gut barrier and provide beneficial effects [16]. To date, the relationship between Akkermansia and epilepsy remains unclear. The study by Olson et al. [6] found that Akkermansia mediated the ketogenic diet-induced protection against 6-Hz-induced seizures in a mouse model of refractory epilepsy. In contrast, Anjiao Peng et al. [17] showed that Verrucomicrobia and Akkermansia were significantly more abundant in the anti-epilepsy drug-resistant group of refractory epilepsy in adults than that in the drug-sensitive group and the healthy control group of normality, which is consistent with our findings and seems to indicate the association of disease symptoms with Akkermansia. Our study also showed that the relative abundance of Akkermansia was higher in patients with WS compared to HCs. This might be possible because the bidirectional effects of Akkermansia mediate different types of epilepsy, and it may or may not be drug-resistant epilepsy. This hypothesis is based on the good response of WS to ACTH and glucocorticoid treatments but poor response to treatment with convention anti-epileptic drugs. On the other hand, some studies also showed adverse effects on the nervous system. Cekanaviciute et al. [18] reported that Akkermansia can induce in vitro differentiation of T lymphocytes toward the pro-inflammatory interferon γ + Th1 phenotype, and an increase in Akkermansia abundance in vivo may directly or indirectly cause severe inflammatory effects in multiple sclerosis, which is similar to our result [18].

For the treatment of WS, pediatricians use ACTH therapy as the first-line of treatment; however, the underlying mechanisms remain unclear. ACTH cannot cross the blood-brain barrier; thus, the therapeutic effects may not be direct [19]. The HPA axis is known to be involved in WS as a potential mechanism, in which an increase in the corticotropin-releasing hormone (CRH) and N-methyl-D-aspartic acid (NMDA) increases the excitability and loss of inhibition [20, 21]. ACTH inhibits the release of CRH by negative feedback, thereby reducing eclampsia of the CRH and neuronal excitability. We hypothesize that exogenous ACTH directly influences the HPA axis dynamics, leading to GM alterations, which could activate the HPA axis by several mediators through the blood-brain barrier. Another hypothesis is that Akkermansia may be correlated to cytokines and immunocytes. In recent years, more and more scholars have discovered the importance of immunological processes in the pathophysiology of epilepsies like WS [22, 23], which might be due to the fact that ACTH mainly exerts anti-inflammatory effects and immune regulation instead of directly alleviating the seizure symptom. Our study showed that Akkermansia is positively correlated to CD3^+^ and CD4^+^ and negatively correlated to TNF-α and CD19^+^. However, the results couldn’t show the details and obvious evidence about the relationship between WS and the immune system, which may help in exploring the underlying mechanism of the therapeutic effects of ACTH in the future.

In regard to the prediction of microbial metabolism, the significant pathway “γ-aminobutyric acid receptor subunit epsilon” seems to be considerably important. The KO analysis showed that GABRE was significantly lower in the BT group than the other two groups. As everyone knows, GABA (γ-aminobutyric acid) is known as a major inhibitory neurotransmitter in the central nervous system, which exerts its effect via ionotropic and metabotropic receptors. These receptors are targeted by many clinically important drugs, such as benzodiazepines that are widely used in the treatment of epilepsy [24]. Thus, GABRE not GABA may play an important role during the ACTH treatment and is worth being evaluated in mouse models.

There were several limitations to our study. First, the sample size was small. Larger sample size would help reach a valid conclusion based on the statistical analysis of the data regarding the relationships between the relative abundances of the GM genera and WS. Second, the treatment of ACTH is a short-term course, followed by treatment with oral steroids for 3 to 6 months, and whether the gut microbiota in these patients with seizure control would change or not should be investigated in future studies. A long-term follow-up is required to comprehensively evaluate the GM alterations in patients with WS. Finally, the underlying mechanism of the ACTH therapy on WS is still unknown. Further research, such as the one using a mouse model, may be used for investigating Akkermansia-related immunological indicators, keeping in mind all the limitations.

## Conclusions

In conclusion, the overall microbial composition differed slightly and the differences existed only in certain genera. The main one was the relative abundance of Akkermansia, which was significantly different between patients with WS and HCs, and between before and after the ACTH therapy. The neuroactive ligand-receptor interaction pathway and GABRE were observed to be significant in the three groups analyzed through functional analysis using PICRUSt2. Most of the patients acquired relief in the clinical symptoms and EEG, as was expected. Meanwhile, changes in some cytokines and immune cells were observed with Akkermansia. Further research is currently being designed to elucidate the relationship between GM and WS completely. The findings of the present study will help in exploring the pathogenesis of WS.

## Methods

### Patients, fecal sample collection, and ACTH therapy

A total of 29 infants (M:F = 20:9) with WS (aged 3–13 months) were enrolled at the Zhejiang University School of Medicine from August 2018 to December 2019 (Table 1). Three typical characteristics, namely, spasms, neurodevelopmental delay, and hypsarrhythmia on EEG, were used for confirming the diagnosis of WS. The control group comprised 29 healthy infants (HCs). The enrolled patients and the healthy controls were of the same age distribution of 3–13 months (Student’s t-test, t = − 0.12 and *P* = 0.91). Besides, the two groups had similar gender proportions (Pearson’s chi-squared test, χ2 = 0 and *P* = 1) and feeding patterns (Fisher’s exact test, *P* = 1). There was no difference (*P* = 1.00 and *P* = 0.850) between the genders in terms of both clinical manifestation and EEG. The patients, as well as the controls, were not given antibiotics or probiotics for at least 3 months before the study. Patient data were collected prospectively, including age, sex, etiology, anti-epileptic drug use, and treatment effects.

The dietary pattern of the HCs, which was divided into three groups (AF/BF/MF: artificial feeding/breastfeeding/mix feeding), was matched with that of the patients with WS. In the meantime, the dietary patterns of the patients were strictly controlled during the ACTH treatment process. However, there were also certain differences, such as the dietary ingredients, between the members in the HC and BT groups. These differences, however, supported our results regarding the statistically significant species. Fecal samples were collected twice (before and after the ACTH therapy) and were transferred to a − 80 °C freezer within 24 h and then sent for research within 7 days of collection.

As per the ACTH therapy, low-dose (20–25 U/day) of natural ACTH extracted from porcine pituitary glands was intravenously injected for > 8 h/day for 2 weeks. The cell cytokines and immunocytes in the blood samples of patients were compared before and after the ACTH treatment. During the treatment period, before the second feces collection, the use of other medicines (such as anti-epileptic drugs, antibiotics, or probiotics) remained fixed (with the same regimen of anti-epileptic drugs throughout and no antibiotics or probiotics started before the second feces collection had been completed).

### DNA extraction, library construction, and sequencing

DNA extraction kit (DP328, Tiangen Biotech Co., Ltd., Beijing, China) was used to extract genomic DNA from the fecal samples as per the manufacturer’s instructions. The hypervariable V3–V4 region of the 16S rRNA gene was amplified using paired primers (341F: 5`-CCTACGGGNGGCWGCAG-3` and 805R: 5`-GACTACHVGGGTATCTAATCC-3`). Then, the Nextera XT Index Kit was used to attach the indices and sequencing adapters. An Agilent SureCycler 8800 Thermal Cycler was used for Polymerase chain reaction (PCR) as per the manufacturer’s protocol. The protocol involved 25 cycles of denaturation at 95 °C for 30 s, annealing at 55 °C for 30 s, and extension at 72 °C for 30 s, followed by a final elongation at 72 °C for 5 min. The DNA was purified using magnetic beads and electrophoresis was conducted for determining the length of the product. Libraries were sequenced and the PE300 raw data in FASTQ format were obtained using the Illumina MiSeq platform (Illumina, Inc., San Diego, CA, USA).

Sequences were further processed to obtain high-quality sequences for analysis to ensure the accuracy of the results. First, primer sequences were removed using Cutadapt (version 1.11) and the resultant paired-end reads were assembled using PANDAseq (version 2.9). Reads with an average quality score of < 20, those with ambiguous bases, the ones with length < 300 or > 480 bp, and chimeric sequences were excluded. Based on 97% similarity, the remaining sequences were clustered into OTUs, of which the representative sequences were assigned to taxa at certain taxonomy levels by RDP-classifier (V1.12) with a confidence cutoff of ≥0.8. Qiime (V1.9) was used to construct the taxonomy profiling table, alpha rarefaction table, and (un)weighted-UniFrac distance matrix.

### Data analysis

Kruskal–Wallis rank test was used to evaluate the differences in the Chao1 index and Shannon index (α diversity), and the relative abundances of GM were compared among HC group (HC), WS patients before (BT), and after (AT) ACTH therapy. Once the Kruskal-Wallis rank test detected differences, further paired tests were performed (Wilcoxon rank-sum test for HC–BT comparison and Wilcoxon signed rank test for BT–AT comparison). R software (version 3.4.3) was used to plot graphs. Subsequently, PICRUSt2 was used to analyze the functional profile predictions of the BT, AT, and HC groups. A principal coordinate analysis (PCoA) was also conducted to analyze the microbial composition.

## Data Availability

The datasets used and/or analyzed during the current study are available from the corresponding author on reasonable request. The raw data of amplicon sequencing have been uploaded on NCBI (SubmissionID: SUB8479680, BioProjectID: PRJNA675180).

## Abbreviations

ACTH: Adrenocorticotropic Hormone
WS: West Syndrome
GM: Gut Microbiota
DNA: Deoxyribonucleic Acid
EEG: Electroencephalograph
HPA Axis: Hypothalamic-pituitary-adrenal Axis
TNF-α: Tumor Necrosis Factor Alpha
IL: Interleukin
IFN-γ: Interferon-γ
CD: Cluster of Differentiation
NK: Natural Killer
GABRE: γ-aminobutyric acid receptor subunit epsilon
KO: KEGG Orthology
GABA: γ-aminobutyric acid
OTUs: Operational Taxonomic Units
PCoA: Principal Coordinate Analysis
PICRUSt: Phylogenetic Investigation of Communities by Reconstruction of Unobserved States
PCR: Polymerase Chain Reaction
RDP: Ribosomal Database Project
AF: Artificial Feeding
BF: Breast Feeding
MF: Mixed Feeding
